# Fabrications of Fully Transparent Gallium Oxide Solar-Blind Photodetectors

**DOI:** 10.3390/nano15211614

**Published:** 2025-10-23

**Authors:** Li-Wen Wang, Tai-Yu Wu, Sheng-Yuan Chu

**Affiliations:** 1 College of Environmental Sciences and Ecology, National University of Tainan, Tainan 70101, Taiwan; 2 Department of Electrical Engineering, National Cheng Kung University, Tainan 70101, Taiwan

**Keywords:** gallium oxide, transparent, solar blind, photodetector

## Abstract

This article presents a remarkable achievement: a gallium oxide-based, non-metallic, fully transparent, and self-powered solar-blind ultraviolet photodetector. We have replaced the traditional metal electrode with gallium-doped zinc oxide (GZO), a transparent conductive oxide, for this transparent purpose. Gallium oxide, a wide-bandgap material suitable for solar-blind detection, is used as the active layer. Glass and natural mica are used for the transparent substrate. The gallium oxide thin film is deposited by RF sputtering at room temperature, with polycrystalline orientation, and the top integrated GZO electrode is also prepared at room temperature using the same technique. This simple two-layer structure device maintains a transmittance of over 88% in the visible spectrum for both substrates, a truly impressive performance. Both glass and mica substrates exhibit self-powered photoresponsivity at 265 nm with responsivities of 8.8 × 10^−9^ and 4.4 × 10^−7^ (A/W), operating with an externally applied voltage of 1 V and boasting a responsivity of around two orders of magnitude with rise/fall times less than 10 s. An X-ray diffractometer, ultraviolet–visible spectroscopy, semiconductor analysis, and a semiconductor electron microscope are used for material analysis and device performance. This article presents a transparent gallium oxide solar-blind photodetector with a simple structure. Our research explains the exceptional transmittance of non-metal electrodes with gallium oxide solar-blind photodetectors, setting a new standard in the field.

## 1. Introduction

Gallium oxide is a well-known wide-bandgap material widely used for power electronics, phosphors, gas sensing, optoelectronic devices, photocatalytic activity, energy storage, low-cost nonvolatile memory functionality, etc. Optoelectronic devices include LEDs, laser diodes, solar cells, and photodetectors [1]. The wavelength range of the solar-blind is between 200 nm and 280 nm and is also called deep ultraviolet. However, most solar radiation is absorbed by the ozone layer. As demand for solar-blind photodetectors increases, they can be used for space detection, military applications such as missile tracking, flame warning, secure communication, high-voltage corona detection, and environmental monitoring. Solar-blind detectors can detect flames in broad daylight or bright industrial environments without false positives from sunlight. With these properties, it can be used on multiple devices. Missile exhaust plumes emit UV radiation in the solar-blind region. Solar-blind photodetectors can detect rocket launches or missile threats even in daylight conditions. The solar-blind UV range can be used for astronomical observations of hot stars, the interstellar medium, and other high-energy phenomena. Solar-blind detectors can monitor high-voltage transmission lines and detect insulation faults or leakage currents during the day. They can be used in the detection of UV emissions from chemical processes, welding arcs, or biological fluorescence without interference from sunlight, and are useful in hazardous gas leak detection, industrial process control, and pollution monitoring. A solar-blind photodetector is based on wide-band materials like gallium oxide (4.6 eV–4.9 eV) [2], AlN [3], AlGaN [4], ZnMgO [5], and diamond [6]. The structure of traditional photodetectors with metal electrodes or a multi-layer structure is based on a PN [7] junction or a Schottky [8] junction, which makes it difficult to achieve transparency. Metal nanowires, such as silver nanowires, have been used to achieve this purpose, sacrificing transmittance to only 55% [9]. Due to the silver nanowire, the bandgap difference between the silver electrode and gallium oxide results in a Schottky contact-based device performance. The transparent electrode, like graphene [10], carbon [11], or transparent conductive [12], has been used as a top electrode. However, a large external voltage of 10 V has been applied. The fully transparent gallium oxide solar blind with a small external voltage has received less attention. However, transparent electronics have a promising future in the next generation, such as bright windows, transparent panels, environmental monitoring, and wearable devices. In this article, we not only present a fully transparent solar-blind photodetector with a low applied voltage and self-powered operation but also inspire optimism about its potential applications in next-generation technologies.

## 2. Materials and Methods

Glass (Corning Eagle Slim, 0.7 mm thick, Corning, NY, USA) was chosen as a transparent device substrate. The glass substrate was cleaned with deionized water (DI water), electronic detergent, acetone, and alcohol with an ultrasonic device. The mica substrate, used to demonstrate the feasibility of flexible applications, was peeled off with tape. The active gallium oxide layer was deposited from a ceramic target (99% gallium oxide, GfE, Nürnberg, Germany) using radio-frequency sputtering at room temperature on both substrates. The electrode comprised gallium-doped zinc oxide (GZO) with an integrated shadow mask, deposited by radio-frequency sputtering using ceramic targets (gallium oxide, 3 wt%; zinc oxide, 97 wt%; GfE, Nürnberg, Germany) at room temperature. The deposition parameter is shown in Table 1. The transmittance was measured using a U-3010 UV-visible spectrophotometer (Hitachi, Japan, Tokyo). X-ray diffraction (XRD) patterns of gallium oxide and GZO thin films were measured using a D8 Advance XRD (Bruker, Billerica, MA, USA) equipped with CuKα radiation (λ = 0.154 nm). The dark and illuminated I-V spectra and time-dependent photoresponse were recorded using a semiconductor analyzer 4155C (Keysight, Santa Rosa, CA, USA) with a 265 nm wavelength under a xenon lamp from photoluminescence (PL) F-7000 (Hitachi, Tokyo, Japan). Light intensity was measured with a PD300 sensor (Ophir, Jerusalem, Israel).

## 3. Results and Discussion

**Figure 1:**
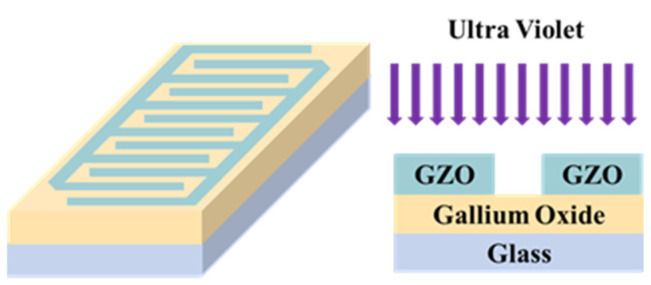
The device structure with the integrated circuit on top.

**Figure 2:**
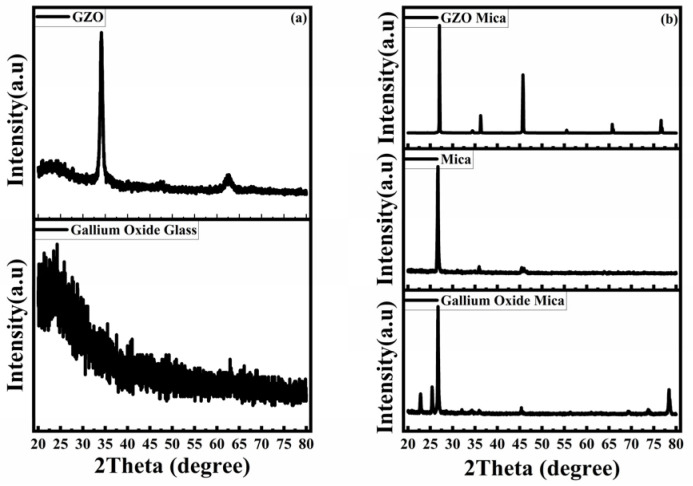
(**a**) The XRD of GZO and gallium oxide thin film on a glass substrate. (**b**) The XRD of the mica substrate, the GZO thin film, and the gallium oxide thin film on the mica substrate.

This transparent gallium oxide-based photodetector with only a two-layer structure is shown in Figure 1. This article presents two substrates, glass and mica; a discussion of the crystal structure is necessary. The top electrode pattern was made with a metal mask with 300 μm interdigitated spacing, 10,150 μm in length, and 9000 μm in width. The XRD measurement result is shown in Figure 2a,b. As the glass substrate is amorphous, the gallium oxide shows an amorphous structure on the glass substrate in Figure 2a. The GZO thin film on the glass substrate shows a (002) orientation, similar to that of zinc oxide [10]. The gallium oxide thin film on the mica substrate shows more crystallization than the glass one. The GZO crystallization was also affected by the substrate, showing multiple peaks in the XRD patterns. According to the XRD results, the crystallization of the electrode and that of the active layer are different.

**Figure 3:**
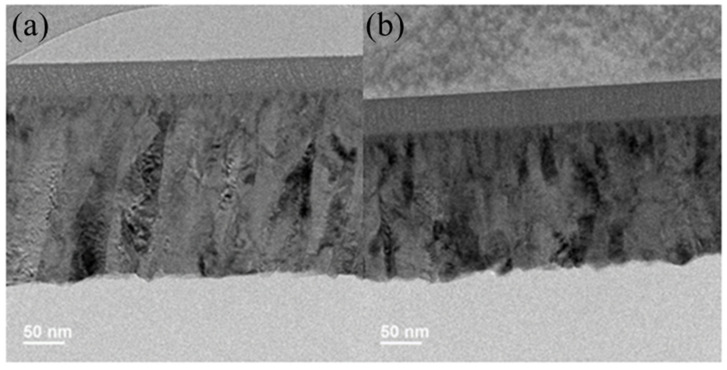
(**a**) Bright-field TEM image shows the cross-section of the gallium oxide and GZO electrode on a glass substrate. (**b**) Bright-field TEM image shows the cross-section of the gallium oxide and GZO electrode on a mica substrate.

**Figure 4:**
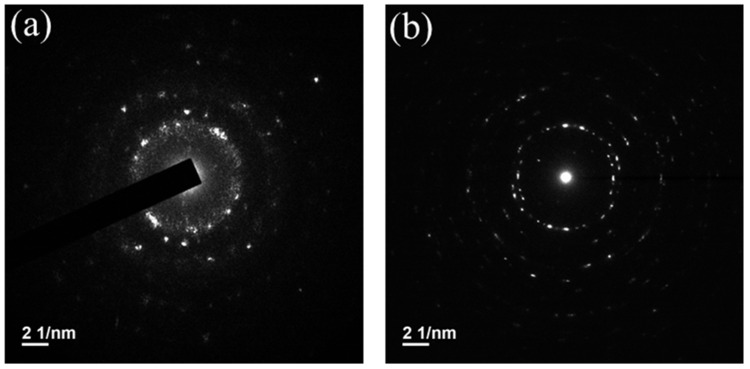
(**a**) High-resolution TEM image of gallium oxide film on the glass substrate. The inset shows the corresponding FFT, which presents a polycrystalline structure. (**b**) High-resolution TEM image of gallium oxide film on the natural mica substrate. The inset shows the corresponding FFT, which presents a polycrystalline structure.

In the XRD measurement, the gallium oxide films on both substrates are amorphous. However, the morphology of gallium oxide thin film, as shown by TEM, is different, as shown in Figure 3a,b and Figure 4a,b. Figure 3a,b show the bright side image by TEM, representing the entire device’s cross-section for both substrates. The gallium oxide and GZO thicknesses were estimated to be 35 nm and 200 nm, respectively, on the glass substrate. On the mica substrate side, the thicknesses of gallium oxide and GZO were 40 nm and 180 nm, respectively. Under the high-resolution top view of gallium oxide shown in Figure 4a,b, in the bright field, randomly arranged atoms can be observed for both substrates. The dark field with the FFT transformer is more obvious with a spotlight [10], which is polycrystalline.

**Figure 5:**
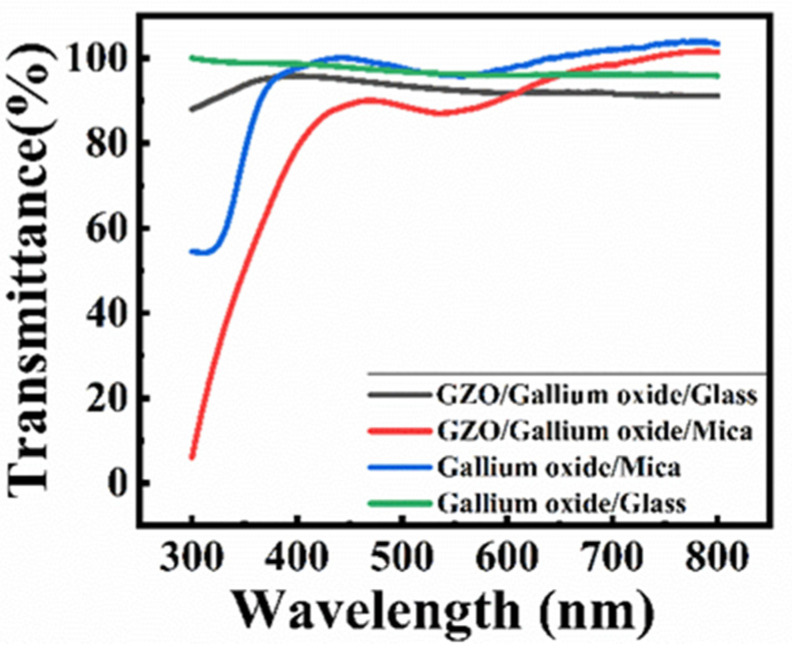
The transmission of gallium oxide thin films on different substrates and devices.

**Figure 6:**
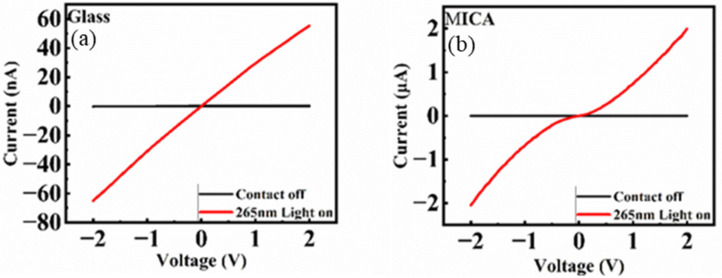
(**a**) The I-V measurement to check the contact between the electrode and the active layer on the glass substrate. (**b**) The I-V measurement to check the contact between the electrode and the active layer on the mica substrate.

As a transparent device, transmittance is a critical characteristic, as shown in Figure 5 by UV-Vis measurement. The visible light spectrum for human eyes is from 380 nm to 700 nm. The transmittance of gallium oxide for both substrates is over 95% in the visible region. With a transparent electrode GZO on top of the device, the transmittance decreases to 88% for mica and 90% for glass. GZO is the electrode material with 97 wt% zinc oxide; the absorption spectrum starts around 360 nm. Because it is not a traditional metal-electrode photodetector, the device’s working principle warrants further discussion. The light intensity of 265 nm is 4 mW/cm^2^. The current–voltage (I-V) curve is used to verify the contact between the electrode and the active layer, as shown in Figure 6a,b. It can be observed that the relation between I-V is a positive linear current, which is a typical ohmic contact [11]. When 265 nm light hits the device, a photocurrent is generated, a phenomenon observed in photoconductive materials. The basic idea of photoconductivity is that when a material absorbs light, the number of free electrons and holes increases within the film, thereby decreasing the resistivity. The photoconductor is difficult to make self-powered.

Instead of a traditional metal-electrode photodetector, the device’s working principle should be further discussed in order to figure out the working principle of this device. Due to the different substrate, the trend of contact is different. The ohmic contact occurs in the low-depletion region, indicating that the thin film contains more defects. As shown by the XRD measurements above, the gallium oxide film on mica exhibits better crystallization, which means fewer defects within the thin film. With fewer defects, the depletion region is wider, and the contact is Schottky. This is why the contact of mica is more like a Schottky contact than that of the films deposited on the glass substrate (Figure 6b has a slight wave compared with the glass one).

**Figure 7:**
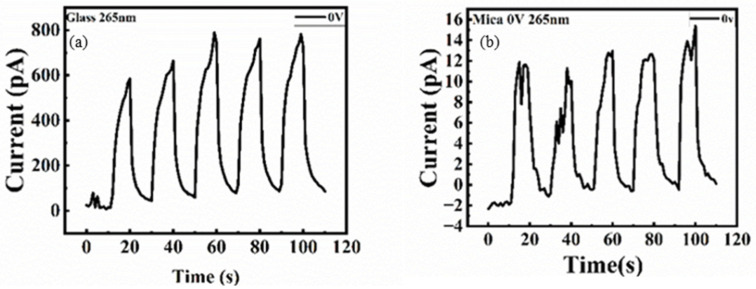
(**a**) One self-powered glass sample under illumination measurement. (**b**) The self-powered mica sample was measured under illumination.

**Figure 8:**
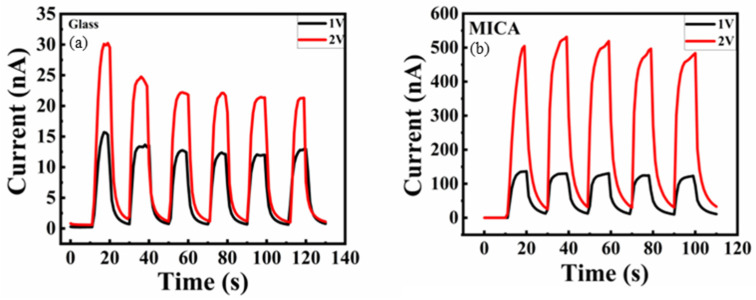
(**a**) The glass sample under illumination measurement with external voltage 1 and 2. (**b**) The mica sample was measured under illumination with external voltage 1 and 2.

A self-powered device has wireless and portable benefits. It is better to have self-power properties. The current–time (I-T) measurements without an external voltage are shown in Figure 7a,b. The light-on and light-off times are 10 s for both. Compared with the mica substrate, the on–off curve is much more stable on the glass substrate, which may be due to its flatness. For both samples, the dark current is quite small, the glass is 56 pA, and the mica is 0.88 pA. Although the self-power response is somewhat small, an external voltage is still required. With the applied voltages of 1 V and 2 V, as shown in Figure 8a,b, the photoresponse is more obvious with external voltage and simultaneous with the self-power one. At higher external voltages, the photo-on-off ratio becomes more pronounced due to greater electron–hole separation. The definitions of rise time and fall time are the times required for the photocurrent to increase from 10% to 90% of the peak value and to decrease from 90% to 10% of the peak value, respectively. The device response time (t_r_) and fall time (t_f_) are presented in Table 2. Under self-power and applied-voltage conduction, the glass sample performs better. This may be due to the roughness of the mica surface.

**Figure 9:**
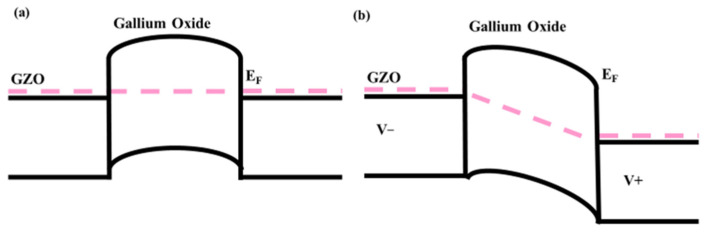
The pink line in both (**a**,**b**) is the Fermi level. (**a**) The bandgap diagram under 0 V. (**b**) The bandgap diagram under an external voltage.

An ohmic contact means the work function difference between the electrode and the active layer is small, so the built-in field is correspondingly small. Figure 9a,b shows the bandgap diagram with or without voltage. The band diagram can elucidate the optical gain of GZO/gallium oxide. Without external voltage, the optical response is based on the difference between the gallium oxide and GZO, which is built in an electric field. As a photoconductor, the bandgap difference is smaller than that of Schottky and P-N junction devices, resulting in poor device performance. The external voltage is needed to improve device performance. As the positive voltage is applied, larger band bending occurs than without the external voltage. The carriers move towards a constant drift. With an external voltage, the electrons and holes separate further, and the dark and light currents increase on both substrates. At an external voltage of 1 V, responsivity and detectivity both increase. As the fully transparent devices have been discussed less, a comparison is shown in Table 2. In Refs. [12,13], carbon and graphene are used as the electrode, and a large external voltage (10 V) is required. With a silver nanowire as the top electrode and a multi-layer structure, self-power properties can be achieved, but at the expense of device transmittance [18]. This work presents a simple, high-transmittance, self-powered ultraviolet photodetector.

## 4. Conclusions

This work presents a fully transparent gallium oxide photoconductor with glass and natural mica substrates. The transmittance for both substrates is over 80%. The fabrication process is straightforward, featuring a simple two-layer structure with all sputtering deposition at room temperature. Without heat treatment, gallium oxide exhibits polycrystalline orientation during thin-film deposition on both glass and mica substrates. In material analysis, the crystalline layer is better on the mica substrate, resulting in improved photoresponse performance. The transparent solar-blind photoconductor can be operated under self-power and with a low external voltage (around 1 to 2 V), achieving an acceptable response time of less than 10 s. If natural mica is replaced with synthetic mica, it would be a good candidate for a transparent, flexible device. This article presents a simple, polycrystalline gallium oxide transparent solar-blind photodetector structure that operates at low applied voltage.

## Figures and Tables

**Table 1:** The deposition parameter of GZO and gallium oxide thin film.

Thin Film	Gas (sccm)	Power (W)	Pressure (mtorr)	Time (min)
GZO	20 Argon	60	2	30
Gallium Oxide	50 Argon	90	5	45

**Table 2:** A comparison of transparent gallium oxide-based photodetectors (GO represents gallium oxide).

Structure	GO Type	Voltage (V)	t_r_/t_f_ (s)	Responsivity (A/W)	Detectivity (Jones)	Transmittance (%)	Reference
GZO/GO/mica	polycrystalline	0	5.7/4.78	8.80 × 10^−9^	9.76 × 10^4^	>80	This work
GZO/GO/glass	polycrystalline	0	6.72/4.7	4.42 × 10^−7^	6.14 × 10^5^	>90	This work
GZO/Go/mica	polycrystalline	1	7/4	8.84 × 10^−5^	7.89 × 10^6^	>80	This work
GZO/GO/glass	polycrystalline	1	1/2.4	1.08 × 10^−2^	1.81 × 10^8^	>90	This work
Graphene/β-GO/	β	10	-	29.8	1.45 × 10^12^	High	[12]
Graphene/PET	Amorphous	10	1.73/3.4	16.34	1.19 × 10^13^	>90 (without electrode)	[13]
Carbon/GO/carbon/Al_2_O_3_	Amorphous	10	1.73/3.4	16.34	1.19 × 10^13^	>90 (without electrode)	[13]
Au/Graphene/β-Ga_2_O_3_/si/in	β	0	<30 ns/<2.24 μs	1.03 × 10^−2^	-	-	[14]
ITO/GO/ITO (array)	ε	10	5.6 ms/7.2 ms	286.2	4.73 × 10^14^	Fully Transparent	[15]
ITO/α-Goβ-Ga_2_O_3_/ITO	Amorphous/β	10	-	74.9	7.4 × 10^15^	Fully Transparent	[16]
Ni/Au/κ-Ga_2_O_3_/	κ-	20	0.81/0.14	703	4.08 × 10^14^	High	[17]

## Data Availability

The original contributions presented in this study are included in the article material. Further inquiries can be directed to the corresponding author.
